# Metagenomic analysis of the gut microbiota of hooded cranes (*Grus monacha*) on the Izumi plain in Japan

**DOI:** 10.1002/2211-5463.13881

**Published:** 2024-09-14

**Authors:** Kosuke Takada, So Nakagawa, Kirill Kryukov, Makoto Ozawa, Tokiko Watanabe

**Affiliations:** ^1^ Department of Molecular Virology, Research Institute for Microbial Diseases Osaka University Japan; ^2^ Department of Molecular Life Science Tokai University School of Medicine Isehara Japan; ^3^ Division of Omics Sciences, Institute of Medical Sciences Tokai University Isehara Japan; ^4^ Division of Interdisciplinary Merging of Health Research, Micro/Nano Technology Center Tokai University Isehara Japan; ^5^ Bioinformation and DDBJ Center National Institute of Genetics Shizuoka Japan; ^6^ Center for Genome Informatics, Joint Support‐Center for Data Science Research Research Organization of Information and Systems Shizuoka Japan; ^7^ Joint Faculty of Veterinary Medicine Kagoshima University Japan; ^8^ Joint Graduate School of Veterinary Medicine Kagoshima University Japan; ^9^ Kagoshima Crane Conservation Committee Izumi Japan; ^10^ Center for Infectious Disease Education and Research Osaka University Japan; ^11^ Center for Advanced Modalities and DDS Osaka University Japan

**Keywords:** ecology, endangered animal, hooded cranes, Metagenome, microbiota

## Abstract

Recent advances in DNA sequencing technology have dramatically improved our understanding of the gut microbiota of various animal species. However, research on the gut microbiota of birds lags behind that of many other vertebrates, and information about the gut microbiota of wild birds such as migratory waterfowl is particularly lacking. Because the ecology of migratory waterfowl (e.g., lifestyle, diet, physiological characteristics) differs from that of other birds, the gut microbiota of migratory waterfowl likely also differs, but much is still unknown. The hooded crane (*Grus monacha*) is an important representative migratory waterbird species and is listed as endangered on the International Union for Conservation of Nature and Natural Resources Red List of Threatened Species. In this study, we analyzed the bacterial and viral microbiota in the gut of hooded cranes by using deep sequencing data from fecal samples of hooded cranes that winter on the Izumi plain in Japan, and found that *Cetobacterium*, *Clupeiformes*, and *Pbunavirus* were clearly present in the fecal samples of hooded cranes. These findings advance our understanding of the ecology of hooded cranes.

## Graphical abstract

We analyzed metagenomic sequencing data obtained from fecal samples of hooded cranes and wild ducks that winter on the Izumi Plain in Japan. Various organisms were identified in each sample, and their diversity differs between the crane and duck groups, suggesting that these two bird species may have distinct gut microbiota.

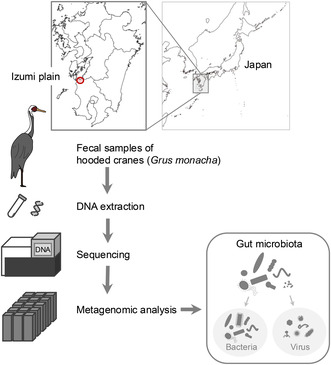

**Abbreviations**
BOLD: barcode of life data
COI: cytochrome oxidase subunit 1
DRA: DDBJ Sequence Read Archive
PC: principal component
PCA: principal component analysis

The gut microbiota of animals varies depending on the lifestyle, diet, environmental, and genome of the host [1]. It also influences the physiological functions and nutritional status (e.g., digestion, absorption, energy metabolism balance, immune defense function, etc.) of each host animal; accordingly, it has attracted the attention of ecologists [2, 3]. To elucidate the composition of the gut microbiota, researchers often analyze bacterial 16S rRNA genes to identify the bacterial species in the microbiota and to estimate their composition based on abundance ratios. Recent advances in DNA sequencing technology have made it possible to simultaneously evaluate not only bacteria but also fungi and DNA viruses by analyzing all DNA sequences contained in a sample and the genetic information of the microbiota in detail [4, 5, 6, 7, 8].

The avian gut microbiota is unique because birds have characteristics and developmental strategies that differ from those of other vertebrates [2]. Yet, the study of the avian gut microbiota has lagged behind that of many other vertebrates, with analyses mainly limited to ornamental and economically important birds (e.g., kakapo, hoatzin, and fowl) [9, 10, 11, 12, 13]. For wild birds, such as migratory waterfowl, the gut microbiota has not been well studied even though their lifestyle, diet, and physiological characteristics are different from those of other birds, and these factors may cause complex changes in their gut microbiota.

The hooded crane (*Grus monacha*) is an important representative migratory waterbird species, listed as endangered on the Red List of the International Union for Conservation of Nature and Natural Resources, with an estimated global population of about 11 600 birds (https://www.iucnredlist.org/species/22692151/93337861). It is an East Asia migratory bird that breeds in forests and meadows in Far East Russia and the northeast of China, but spends the winter in the wetlands of Korea, Japan, and the middle and lower reaches of the Yangtze River in China [14]. Hooded cranes spend a relatively long time in wintering and breeding grounds, with a comparatively shorter time at stopover sites [15]. In winter, the hooded crane population in Japan is about 10 500, and most of these birds are found on the Izumi plain, Kagoshima, in southernmost Kyushu, Japan. Hooded cranes are omnivorous, feeding on plant roots, insects, and amphibians. In their wintering grounds (e.g., Izumi plain), they feed on the second ears of rice in paddy fields, wheat, and sardines.

In this study, we analyzed the bacterial and viral microbiota in the gut of hooded cranes by analyzing nucleotide sequence data obtained by deep sequencing fecal samples from hooded cranes that wintered in the Izumi plain, Japan, to determine the composition of bacterial and viral species of the microbiota. In addition, we used the nucleotide sequence data to gain insights into the diet of the hooded crane. Our findings advance our understanding of the ecology of hooded cranes and may be useful in efforts to conserve this endangered species.

## Materials and methods

### Sample collection

On November 20, 2016, 10 fecal samples were collected in the Izumi plain, Japan. Feces were collected with disposable plastic spoons and placed into sterile 2‐mL sample tubes, which were kept on ice. The fecal samples were transported to the laboratory in a cooler with ice packs for storage at −80 °C.

### DNA extraction and sequencing

DNA was extracted from each fecal sample using a QIAamp DNA Stool Mini Kit (Qiagen, Hilden, Germany) according to the manufacturer's protocol. Library construction and deep sequencing were performed on the paired‐end Illumina HiSeq sequencing platform (HiSeq 2500, 2 × 150 bp) by GENEWIZ (South Plainfield, NJ, USA). The obtained sequences were registered in the DDBJ Sequence Read Archive (DRA) (accession numbers DRR173096–DRR173101 for crane and DRR279219–DRR279222 for duck).

### Identification of host (bird) species

To identify the host origin of the fecal samples, 34 400 bird barcode sequences of the mitochondrial gene, cytochrome oxidase subunit 1 (COI), were downloaded from the Barcode of Life Data (BOLD) System (July 5, 2017) (http://www.boldsystems.org). Using the BOLD database as a target, we conducted a homology search using blastn version 2.6.0 [16, 17] with the entire sequences from each sample as a query. The sensitivity parameters of blastn used for the analysis were as follows: −evalue 1e‐10 −dbsize 3 200 000 000 [18]. Since BLAST e‐value calculations depend on the database size, and we frequently use databases of different sizes in our experiments, we configured the same effective database size in our blast runs to have consistent e‐value cut‐offs across different experiments. The effective database size selected was subjective and based on the size of the human genome. The hits were then filtered to retain only those with a bit score of at least 210 [18], and species associated with sequences showing the highest bit score were assumed to be the host species from which each feces sample was obtained.

### DNA sequencing data analysis

To identify the species composition of each fecal sample, we analyzed the DNA sequencing reads as follows and previously described [19]. First, all the reads were compared with representative genomes of eukaryotes, bacteria, archaea, and viruses (Table S1) obtained from GenomeSync database (http://genomesync.org) using minimap2 version 2.17 [20] with the default option. The best‐hit reads assigned to each species were obtained. However, due to the limitation of the sequence read length (150 bp), we counted each read at the genus level rather than the species level. To identify each species in a given sample, we performed sequence assembly for each sample using spades version 3.14.0 [21] with the following options: −k 21,33,55,77,99,127. Principal component analysis (PCA) was conducted using the prcomp function in the R version 4.0.3 (2020‐10‐10). We evaluated the diversity of each sample by calculating the alpha diversity by using Shannon's Diversity Index and the beta diversity by using the Jaccard Index of Dissimilarity. The source code is available at https://biokirr.com/Supporting‐Data/Diversity‐Scripts/.

## Results

### Identification of bird species based on the DNA sequence of fecal samples

We extracted DNA from 10 waterfowl‐derived fecal samples collected in the Izumi plain, Japan. For each sample, we conducted metagenomic sequencing, the results of which are summarized in Table 1. We first identified the waterfowl species from which each fecal sample was taken (see also Materials and methods). Of the 10 samples analyzed, six samples (#1 to #6) were determined to be derived from hooded crane (*Grus monacha*) (Table 1) as we previously reported [18]. The remaining four species were identified as wild ducks belonging to the genus *Anas*: two samples (#7 and #9) were determined to be Eurasian wigeon (*Anas penelope*), one sample (#10) was determined to be northern pintail (*Anas acuta*) (Table 1), and the last sample (#8) could not be categorized to the species level and was classified as either mallard (*Anas platyrhynchos*) or spot‐billed duck (*Anas zonorhyncha*) (Table 1). In summary, we found that the fecal samples we collected included six hooded crane feces samples and four various wild duck samples.

**Table 1:** Bird species identified in each feces sample.

ID	Sample_number	Common name	Scientific name	Total reads
#1	S85	Hooded crane	*Grus monacha*	59 812 720
#2	S86	Hooded crane	*Grus monacha*	72 196 978
#3	S87	Hooded crane	*Grus monacha*	58 869 812
#4	S89	Hooded crane	*Grus monacha*	76 627 514
#5	S90	Hooded crane	*Grus monacha*	64 758 590
#6	S91	Hooded crane	*Grus monacha*	60 560 614
#7	S88	Eurasian wigeon	*Anas penelope*	70 800 830
#8	S92	Mallard or/and spot‐billed duck	*Anas platyrhynchos* or/and *Anas zonorhyncha*	60 633 814
#9	S93	Eurasian wigeon	*Anas penelope*	71 222 608
#10	S94	Northern pintail	*Anas acuta*	59 872 248

### Identification of DNA sequencing reads contained in waterfowl fecal samples

**Fig. 1:**
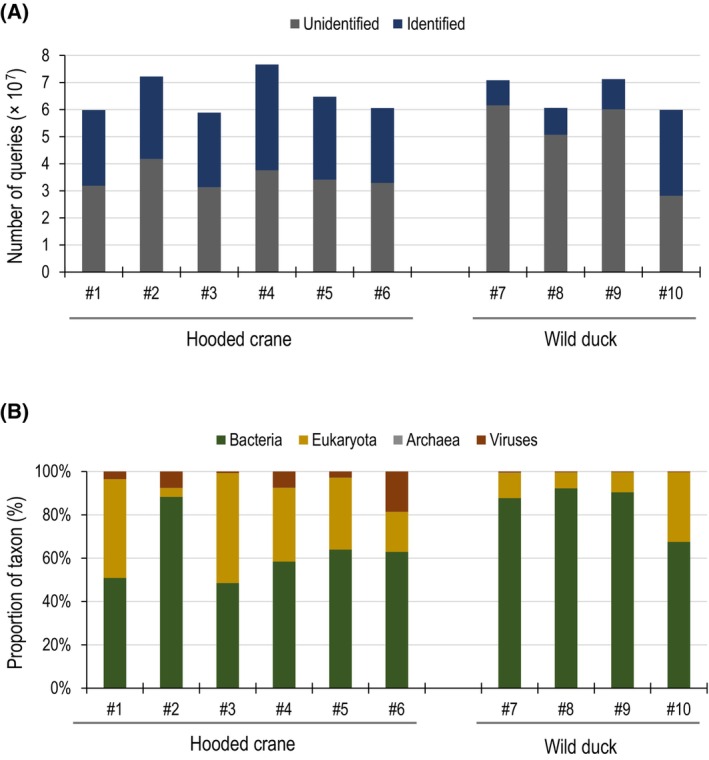
Identification of DNA sequencing reads contained in fecal samples of hooded crane and wild duck. (A) The number of sequence reads in each sample are shown. Reads identified as eukaryotic, bacterial, archaeal, or viral sequences are shown in blue, and unidentified reads are shown in gray. (B) Breakdown of the reads identified as eukaryotic, bacterial, archaeal, or viral sequences. Bacterial, eukaryotic, archaeal, and viral populations are shown in green, yellow, gray, and brown, respectively.

To examine the diet and intestinal microbiota of hooded crane and wild duck, we analyzed the sequence reads obtained from the 10 fecal samples by using the minimap2 program against eukaryote, bacteria, archaea, and viral genome sequences obtained from the GenomeSync database [22] (http://genomesync.org, summarized in Table S1). The average percentage of sequence reads identified as any sequence of eukaryotes, bacteria, archaea, or viruses was 46.6% (#1, 46.7%; #2, 42.2%; #3, 46.8%; #4, 50.9%; #5, 47.4%; #6, 45.6%) and 24.5% (#7, 13.0%; #8, 16.4%; #9, 15.7%; #10, 52.9%) in the hooded crane and wild duck samples, respectively (Fig. 1A). For each sample, we further analyzed the fraction of organisms detected.

Percentages in each sample were analyzed for readings identified as sequences of bacteria, eukaryotes, archaea, or viruses. Bacteria were the major organisms in all samples examined in this study for both the hooded crane and wild duck samples. For the six hooded crane samples, bacteria accounted for an average of 62.17% of the identified reads (#1, 50.88%; #2, 88.34%; #3, 48.53%; #4, 58.45%; #5, 63.94%; #6, 62.88%), whereas for the four wild duck samples bacteria accounted for an average of 84.47% of reads (#7, 87.71%; #8, 92.24%; #9, 90.40%; #10, 67.53%) (Fig. 1B and Table S2). Eukaryotes accounted for an average of 31.02% of identified reads for the hooded crane samples (#1, 45.58%; #2, 4.10%; #3, 50.73%; #4, 34.02%; #5, 33.20%; #6, 18.51%), and an average of 15.12% for the wild duck samples(#7, 11.69%; #8, 7.35%; #9, 9.27%; #10, 32.17%) (Fig. 1B and Table S2). Archaea accounted for 0.02% of the hooded crane samples (#1, 0.02%; #2, 0.02%; #3, 0.03%; #4, 0.02%; #5, 0.02%; #6, 0.02%) and 0.08% of the wild duck samples (#7, 0.19%; #8, 0.06%; #9, 0.07%; #10, 0.02%) (Fig. 1B and Table S2) The proportion of reads corresponding to viruses differs greatly: an average of 6.78% for the hooded crane samples (#1, 3.52%; #2, 7.54%; #3, 0.71%; #4, 7.51%; #5, 2.84%; #6, 18.59%) and 0.33% for the wild duck samples (#7, 0.41%; #8, 0.35%; #9, 0.26%; #10, 0.29%) (Fig. 1B and Table S2).

### Analysis of intestinal microbiota in hooded crane feces

**Fig. 2:**
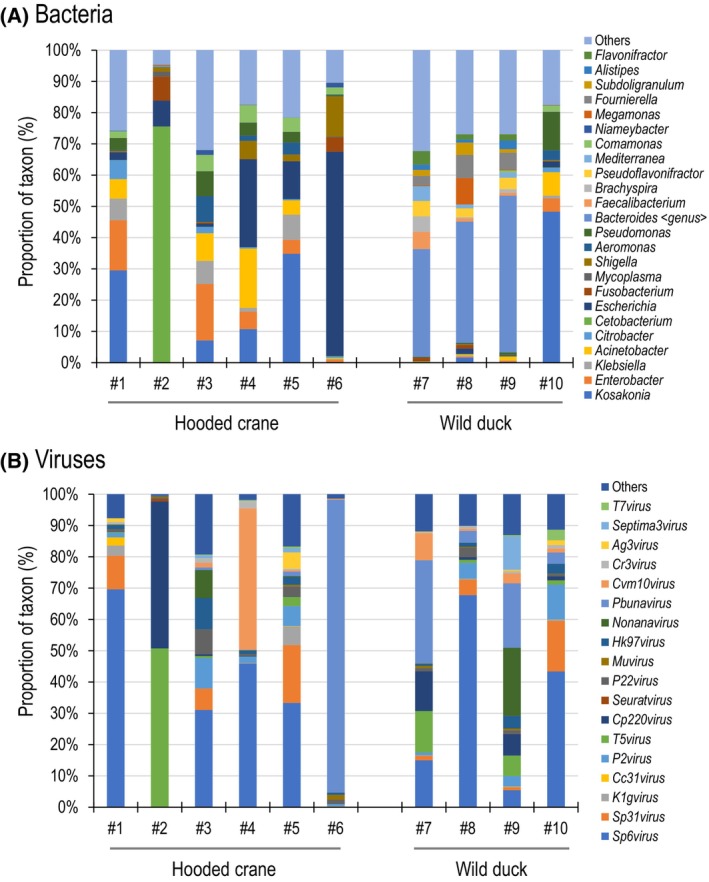
Analysis of intestinal microbiota of hooded cranes and wild ducks. (A) Proportion of the major bacterial genera in each sample. The top five bacterial genera in each sample are shown. (B) Proportion of the major viral genera in each sample. The top five viral genera in each sample are shown.

Bacteria sequences predominated in all samples examined in this study. To investigate the intestinal microbiota of hooded cranes and wild ducks, we analyzed the bacteria and archaea sequence reads contained in the 10 fecal samples. We analyzed the reads identified as bacteria at the genus level and obtained a total of 24 genera (Fig. 2A and Table 2 show the top five genera with the number of reads identified as bacteria in each sample). The proportion of identified bacteria varied greatly depending on the samples. For both hooded crane and wild duck samples, the genus *Kosakonia* was ranked high among the reads identified as bacteria in multiple samples. Among the reads identified as bacteria, the genus *Kosakonia* accounted for an average of 13.74% (#1, 29.53%; #2, 0.03%; #3, 7.12%; #4, 10.72%; #5, 34.82%; #6, 0.23%) among the hooded crane samples and 12.56% (#7, 0.09%; #8, 1.59%; #9, 0.28%; #10, 48.30%) among the wild duck samples. The genus *Escherichia*, which ranked high in multiple hooded crane samples, accounted for an average of 19.56% (#1, 2.47%; #2, 8.28%; #3, 1.01%; #4, 28.08%; #5, 12.08%; #6, 65.43%) of the reads identified as bacteria. In contrast, in the wild duck samples, the genus *Escherichia* accounted for an average of 1.01% of the reads identified as bacteria, representing a low level for all samples examined: #7, 0.08%; #8, 1.75%; #9, 0.30%; #10, 1.91%. The genus *Bacteroides*, which rankled high in multiple wild duck samples, accounted for an average of 30.89% (#7, 34.48%; #8, 38.78%; #9, 50.17%; #10, 0.13%) of the reads identified as bacteria. Among the hooded crane samples, the genus *Bacteroides* accounted for an average of 0.06% (#1, 0.01%; #2, 0.30%; #3, 0.02%; #4, 0.02%; #5, 0.02%; #6, 0.02%) of the reads identified as bacteria.

**Table 2:** Percentage of taxa in the top five of the bacterial reads identified in each sample. The taxon ranking of the bacteria identified in each sample is shown in parentheses. Only the top 1 to 20 rankings are shown.

Taxon name	Hooded crane													Wild duck								
	#1		#2		#3		#4		#5		#6		Average	#7		#8		#9		#10		Average
*Kosakonia*	29.53	(1)	0.03		7.12	(6)	10.72	(3)	34.82	(1)	0.23	(19)	13.74	0.09		1.59	(9)	0.28		48.30	(1)	12.56
*Enterobacter*	16.04	(2)	0.01		18.04	(1)	5.56	(6)	4.45	(6)	0.69	(8)	7.47	0.03		0.30		0.32		4.24	(4)	1.22
*Klebsiella*	6.92	(3)	0.02		7.40	(5)	1.22	(12)	8.06	(3)	0.28	(17)	3.98	0.02		0.36		0.09		0.90	(12)	0.34
*Acinetobacter*	6.25	(4)	0.10		8.86	(2)	19.03	(2)	4.61	(4)	0.32	(14)	6.53	0.18		0.39		1.24	(10)	7.52	(3)	2.33
*Citrobacter*	6.05	(5)	0.01		1.98	(11)	0.43	(15)	0.37		0.40	(11)	1.54	0.01		0.09		0.05		1.38	(8)	0.38
*Cetobacterium*	0.05		75.41	(1)	0.07		0.02		0.03		0.15		12.62	0.09		0.04		0.05		0.17		0.09
*Escherichia*	2.47	(8)	8.28	(2)	1.01	(15)	28.08	(1)	12.08	(2)	65.43	(1)	19.56	0.08		1.75	(7)	0.30		1.91	(6)	1.01
*Fusobacterium*	0.04		7.66	(3)	0.33		0.12		0.04		4.63	(3)	2.14	1.12	(14)	1.09	(13)	0.02		0.05		0.57
*Mycoplasma*	0.01		1.56	(4)	0.01		0.01		0.02		0.22	(20)	0.30	0.02		0.01		0.02		0.01		0.02
*Shigella*	0.43		1.47	(5)	0.18		5.78	(4)	2.11	(11)	12.93	(2)	3.82	0.01		0.31		0.06		0.37	(20)	0.19
*Aeromonas*	0.31		0.01		8.26	(3)	1.70	(9)	3.84	(7)	0.21		2.39	0.04		0.15		0.05		3.13	(5)	0.84
*Pseudomonas*	3.83	(6)	0.03		7.98	(4)	4.17	(7)	3.42	(9)	0.33	(13)	3.29	0.20		0.24		0.77	(19)	12.29	(2)	3.38
*Bacteroides* <genus>	0.01		0.30	(9)	0.02		0.02		0.02		0.02		0.06	34.48	(1)	38.78	(1)	50.17	(1)	0.13		30.89
*Faecalibacterium*	0.00		0.00		0.00		0.00		0.00		0.00		0.00	5.52	(2)	1.06	(14)	0.90	(15)	0.00		1.87
*Brachyspira*	0.00		0.00		0.00		0.00		0.00		0.00		0.00	4.95	(3)	0.37		1.21	(12)	0.01		1.64
*Pseudoflavonifractor*	0.00		0.01		0.00		0.00		0.00		0.00		0.00	4.93	(4)	2.81	(5)	3.67	(3)	0.01		2.86
*Mediterranea*	0.00		0.01		0.00		0.00		0.00		0.00		0.00	4.70	(5)	1.19	(12)	1.65	(6)	0.00		1.89
*Comamonas*	2.11	(9)	0.04		5.23	(7)	5.56	(5)	4.51	(5)	2.26	(4)	3.28	0.03		0.08		0.68		1.85	(7)	0.66
*Niameybacter*	0.16		0.05		1.54	(12)	0.09		0.01		1.43	(5)	0.55	0.01		0.00		0.00		0.01		0.00
*Megamonas*	0.00		0.19	(11)	0.01		0.05		0.01		0.03		0.05	0.24		8.44	(2)	0.22		0.07		2.24
*Fournierella*	0.00		0.14	(12)	0.00		0.00		0.00		0.00		0.02	3.04	(7)	7.39	(3)	5.46	(2)	0.07		3.99
*Subdoligranulum*	0.00		0.01		0.00		0.00		0.00		0.00		0.00	1.94	(9)	3.94	(4)	1.12	(13)	0.01		1.75
*Alistipes*	0.00		0.00		0.00		0.00		0.00		0.00		0.00	1.64	(10)	0.97	(17)	2.75	(4)	0.00		1.34
*Flavonifractor*	0.00		0.00		0.00		0.00		0.00		0.00		0.00	4.36	(6)	1.72	(8)	2.03	(5)	0.01		2.03
Others	25.77		4.66		31.95		17.44		21.59		10.43		18.64	32.30		26.90		26.89		17.56		25.91

Although few sequence reads were detected as archaea across all samples, the genus *Methanoculleus* was the major genus in both the hooded crane and wild duck fecal samples (Fig. 1B, Tables S2 and S3). Indeed, the hooded crane samples showed an average of 92.27% *Methanoculleus* reads (#1, 90.45%; #2, 84.17%; #3, 93.17%; #4, 94.62%; #5, 96.65%; #6, 96.56) (Fig. S1A and Table S3). Similarly, in the wild duck samples, the genus *Methanoculleus* accounted for an average of 88.07% of archaea reads (#7, 86.92%; #8, 82.01%; #9, 88.43%; #10, 94.90%) (Fig. S1A and Table S3). Other reads corresponding to archaea were limited and little diversity in archaea was detected (Fig. S1A and Table S3).

In addition, we examined reads identified as viruses in each sample. Note that RNA virus would not be detected because DNA was extracted from the fecal samples. The top five genera identified as viruses in each sample were all bacteriophages (Fig. 2B and Table 3). The genus *Sp6virus* was ranked high in multiple hooded crane and wild duck samples. Among the hooded crane samples, the genus *Sp6virus* accounted for an average of 30.00% (#1, 69.64; #2, 0.01%; #3, 31.09%; #4, 45.93%; #5, 33.34%; #6, 0.01%) of virus reads. Among the wild duck samples, the genus *Sp6virus* accounted for an average of 32.89% of virus reads (#7, 15.00%; #8, 67.76%; #9, 5.42%; #10, 43.36%). The genus *Pbunavirus* accounted for an average of 15.25% (#7, 33.00%; #8, 3.79%; #9, 20.58%; #10, 3.63%) in wild duck samples, whereas the genus *Pbunavirus* made up 1.5% or less of virus reads among the hooded crane samples, except for #6 (#6, 93.57%). Thus, high proportions of bacteriophage were found in the DNA in the fecal samples of hooded cranes and wild duck.

**Table 3:** Percentage of taxa in the top five of the viral reads identified in each sample. The taxon rankings of the viruses identified in each sample are shown in parentheses. Only the top 1 to 20 rankings are shown. N.D. indicates that no virus reads were detected.

Taxon name	Hooded crane													Wild duck								
	#1		#2		#3		#4		#5		#6		Average	#7		#8		#9		#10		Average
Sp6virus	69.64	(1)	0.01	(15)	31.09	(1)	45.93	(1)	33.34	(1)	0.01		30.00	15.00	(2)	67.76	(1)	5.42	(6)	43.36	(1)	32.89
Sp31virus	10.73	(2)	0.00		6.82	(6)	0.03		18.48	(2)	0.00		6.01	1.41	(7)	4.93	(3)	0.91	(13)	16.18	(2)	5.86
K1gvirus	3.25	(3)	0.01	(19)	0.02		0.15	(17)	5.90	(4)	0.35	(8)	1.61	0.19		0.34	(20)	0.26		0.48		0.32
Cc31virus	2.59	(4)	0.00		0.02		0.01		0.16		0.00		0.46	0.05		0.03		0.08		0.03		0.05
P2virus	1.52	(5)	0.18	(8)	9.69	(3)	2.01	(4)	6.36	(3)	0.65	(5)	3.40	0.84	(10)	4.99	(2)	3.32	(9)	11.06	(3)	5.05
T5virus	0.21		50.57	(1)	0.65		0.02		2.96	(8)	0.03	(14)	9.07	13.27	(3)	1.00	(10)	6.54	(5)	1.40	(10)	5.55
Cp220virus	0.06		46.94	(2)	0.57		0.17	(15)	0.14		0.02	(18)	7.98	12.67	(4)	0.85	(12)	6.85	(4)	1.28	(12)	5.41
Seuratvirus	0.00		0.79	(3)	0.01		0.00		0.00		0.00		0.13	N.D.		0.03		0.05		0.01		0.02
P22virus	0.88	(11)	0.41	(4)	7.93	(5)	0.56	(6)	3.19	(7)	1.36	(3)	2.39	1.07	(8)	2.99	(5)	1.14	(12)	0.68	(17)	1.47
Muvirus	0.02		0.34	(5)	0.03		0.05	(20)	0.49		1.57	(2)	0.42	0.70	(11)	0.32		0.59	(17)	0.13		0.43
Hk97virus	1.11	(9)	0.18	(7)	10.03	(2)	1.00	(5)	2.48	(10)	0.69	(4)	2.58	0.53	(15)	1.21	(7)	4.03	(7)	3.14	(6)	2.23
Nonanavirus	0.26	(20)	0.00		8.93	(4)	0.18	(14)	0.34		0.00		1.62	0.19		0.05		21.79	(1)	0.01		5.51
Pbunavirus	0.19		0.06	(10)	0.84	(19)	0.19	(12)	1.42	(13)	93.57	(1)	16.05	33.00	(1)	3.79	(4)	20.58	(2)	3.63	(4)	15.25
Cvm10virus	0.07		0.02	(14)	1.50	(10)	45.16	(2)	0.47		0.37	(7)	7.93	8.62	(5)	0.62	(15)	3.17	(10)	1.29	(11)	3.42
Cr3virus	0.63	(12)	0.00		1.32	(13)	2.10	(3)	0.43		0.00		0.75	0.33	(18)	0.80	(13)	0.72	(16)	1.08	(13)	0.73
Ag3virus	1.12	(8)	0.00		0.10		0.12	(18)	5.23	(5)	0.00		1.10	0.23		0.11		0.43		1.52	(9)	0.57
Septima3virus	0.05		0.00		0.98	(16)	0.26	(9)	1.28	(14)	0.00		0.43	N.D.		0.01		10.66	(3)	0.02		2.67
T7virus	0.02		0.00		0.21		0.21	(10)	0.62	(19)	0.00		0.18	N.D.		0.04		0.43	(20)	3.37	(5)	0.96
Others	7.65		0.50		19.27		1.83		16.69		1.37		7.89	11.92		10.12		13.04		11.33		11.60

### Prediction of the feeding habits of hooded cranes

**Fig. 3:**
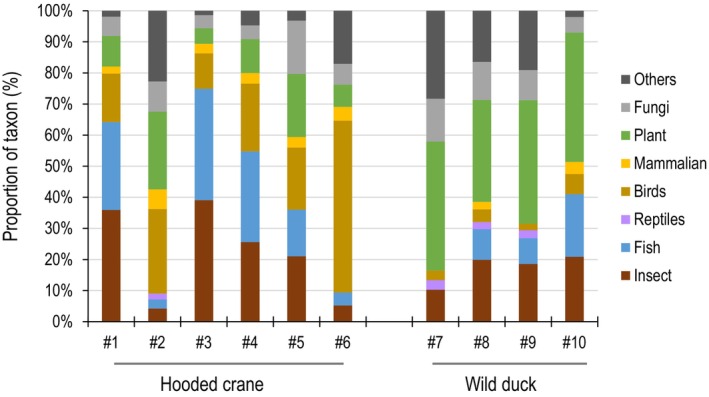
Reconstructed composition of the recent diet of hooded crane and wild duck based on fecal samples. The proportion for each group (insect, fish, reptiles, birds, mammalian, plant, fungi, and the other) was further classified with the eukaryotic reads.

DNA sequencing reads identified as eukaryotes in the fecal samples were likely derived from the food eaten by the waterfowl. We therefore predicted the feeding habits of the waterfowl, especially the hooded cranes, by analyzing the reads identified as eukaryotes. We categorized all eukaryote reads into eight groups (insect, fish, reptiles, birds, mammalian, plant, fungi, and other). We further examined the eukaryote reads at the order level, and the major order accounting for more than 1% of all eukaryote reads in each sample is shown in Table 4, and its proportion in each sample is shown in Fig. 3. We found that the proportion of reads corresponding to insects was similar between hooded crane and wild duck (an average of 21.9% and 17.4% of eukaryote sequences in hooded crane and wild duck samples, respectively). The proportion of reads corresponding to fishes in the hooded crane samples (19.2% on average) was higher than that in the wild duck samples (9.6% on average). Conversely, the proportion of reads corresponding to plants in the hooded crane samples (13.0% on average) was lower than that in the wild duck samples (38.9% on average). Interestingly, *Squamata* (reptile) reads were detected in several fecal samples of both species (#2, #7, #8 and #9), albeit at low levels (Table 4 and Fig. 3).

**Table 4:** Classification of major eukaryotes detected in hooded crane or wild duck samples. Among the taxa identified as eukaryotes in each sample, taxons accounting for more than 1% of all eukaryotes are shown. ‘–’ Indicates that there is no corresponding taxon.

ID	Host	Taxonomy accounted for more than 1% of the reads identified as eukaryotes in each sample							
		Insect	Fish	Reptiles	Birds	Mammalian	Plant	Fungi	Other
#1	Hooded crane	Lepidoptera, Hymenoptera	Clupeiformes	–	Rheiformes, Gruiformes	Cetartiodactyla	Poales, Fabales, Solanales, Malvales	Acytosteliales, Peronosporales	Rhabditida
#2		Lepidoptera, Blattodea	Gadiformes	Squamata	Gruiformes	Carnivora, Chiroptera	Solanales, Poales, Fagales, Fabales	Acytosteliales, Hypocreales, Dictyosteliales, Saccharomycetales	Trichinellida, Rhabditida, Longamoebia
#3		Lepidoptera, Hymenoptera	Clupeiformes	–	Rheiformes	Cetartiodactyla	Poales, Solanales, Malvales	Peronosporales, Acytosteliales	Rhabditida
#4		Lepidoptera, Hymenoptera	Clupeiformes	–	Gruiformes, Rheiformes	Cetartiodactyla	Fabales, Poales, Solanales	Peronosporales, Acytosteliales	Trichinellida, Rhabditida
#5		Lepidoptera, Hymenoptera	Clupeiformes	–	Gruiformes, Rheiformes	Cetartiodactyla	Poales, Solanales	Acytosteliales, Peronosporales, Pucciniales	Rhabditida, Trichinellida
#6		Lepidoptera, Hymenoptera	Clupeiformes	–	Gruiformes, Rheiformes	Carnivora	Solanales, Fagales, Poales	Acytosteliales, Dictyosteliales	Trichinellida, Rhabditida, Adinetida
#7	Wild duck	Lepidoptera, Blattodea, Diptera, Isopoda	–	Squamata	Ciconiiformes, Gruiformes	–	Solanales, Poales, Fabales, Rosales, Lamiales	Hypocreales, Saccharomycetales, Mucorales, Agaricales, Peronosporales, Eurotiales, Ceraceosorales	Rhabditida, Longamoebia, Trichinellida, Cyclophyllidea, Decapoda, Kinetoplastida, Actiniaria, Plagiorchiida
#8		Lepidoptera, Blattodea, Hymenoptera, Diptera	Clupeiformes	Squamata	Rheiformes, Anseriformes	Carnivora	Solanales, Poales, Fabales	Acytosteliales, Peronosporales, Hypocreales, Saccharomycetales, Mucorales	Rhabditida, Trichinellida, Longamoebia, Cyclophyllidea
#9		Lepidoptera, Blattodea, Hymenoptera, Diptera	Clupeiformes	Squamata	Rheiformes	–	Solanales, Poales, Fabales, Charales, Rosales	Hypocreales, Peronosporales, Saccharomycetales, Mucorales, Mortierellales	Rhabditida, Longamoebia, Decapoda, Cyclophyllidea, Trichinellida
#10		Lepidoptera, Hymenoptera	Clupeiformes	–	Rheiformes	Cetartiodactyla	Poales, Fabales, Solanales	Peronosporales	Rhabditida

We further analyzed the reads at the order level corresponding to insects, fishes, and plants. Among the insects, the order *Lepidoptera*, which includes butterflies and moths, was detected in all hooded crane and wild duck samples. The order *Hymenoptera*, including ants, bees, sawflies, and wasps, was detected in five of the six hooded crane samples, and in three of the four wild duck samples. The order *Blattodea*, which includes cockroaches and termites, was detected in three of the four wild duck samples, but in only one of the six hooded crane samples. The order *Diptera*, which includes flies, was detected in three of the four wild duck samples, but not in any of the hooded crane samples. Among the fish reads, the order *Clupeiformes*, including anchovies, herrings, and sardines, was detected in five of the six hooded crane samples, and in three of the four wild duck samples. The order *Gadiformes* (e.g., cod and pollack) was detected in one of the six hooded crane samples, but not detected in wild duck samples. For plants, the order *Poales*, including rice and wheat, was detected in all hooded cranes samples and wild duck samples. The order *Solanales*, which includes potato, eggplants, and tomato, was detected in all hooded cranes samples and wild duck samples. The order *Fabales* (e.g., legumes, peas, and soybeans) was detected in all wild duck samples, and in three of the six hooded cranes samples. The order *Malvales*, including daphnes, hibiscus, and hollyhocks, was detected in two of the six hooded cranes samples, but not detected in wild duck samples. The order *Fagales*, which includes acorns, oaks, and walnuts, was detected in two of the six hooded crane samples, but not in wild duck samples. The order *Rosales*, including apples, roses, and strawberries, was detected in two of the four wild duck samples, but not in any of the hooded crane samples. The order *Charales*, including stoneworts, was detected in one of the four wild duck samples, but was not detected in the hooded crane samples.

Four orders*—Lepidoptera* of insects, *Clupeiformes* of fishes, and *Poales* and *Solanales* of plants—were found in all the hooded crane and wild duck samples. We also found that the proportion of reads corresponding to each order varied greatly depending on the samples for both species. *Lepidoptera* accounted for on average 12.21% of eukaryote reads in the hooded cranes samples (#1, 19.45%; #2, 2.13%; #3, 22.11%; #4, 13.32%; #5, 12.94%; #6, 3.31) and on average 8.35% of the eukaryote reads in the wild duck samples (#7, 3.56%; #8, 7.32%; #9, 7.46%; #10, 11.21%). The hooded crane samples contained 17.09% *Clupeiformes* on average (#1, 26.47%; #2, 0.41%; #3, 32.95%; #4, 25.76%; #5, 13.34%; #6, 3.63) compared with 9.78% on average in the wild duck samples (#7, 0.38%; #8, 7.64%; #9, 6.45%; #10, 17.34%). On average, 5.16% (#1, 5.35%; #2, 3.39%; #3, 1.93%; #4, 2.56%; #5, 16.42%; #6, 1.30) of eukaryote reads belonged to *Poales* among the hooded crane samples, whereas on average 11.23% (#7, 6.15%; #8, 7.89%; #9, 5.40%; #10, 31.56%) of eukaryote reads belonged to *Poales* among the wild duck samples. The percentage of *Solanales* averaged 12.83% for wild duck samples (#7, 24.48%; #8, 16.44%; #9, 18.04%; #10, 1.43%) and 3.77% for hooded crane samples (#1, 1.26%; #2, 13.13%; #3, 1.37%; #4, 1.83%; #5, 1.67%; #6, 3.37).

### Comparison of organisms in each fecal sample

**Fig. 4:**
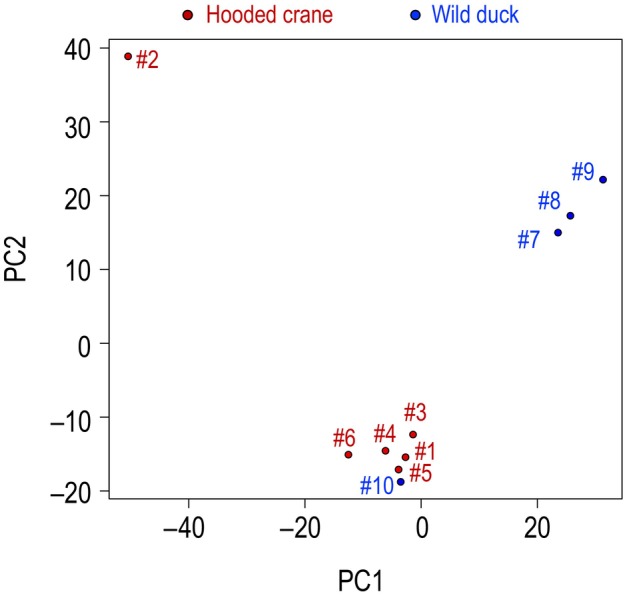
Principal component analysis (PCA) of each of the 10 samples. The fraction of sequence reads corresponding to the order of eukaryotes or genus bacteria, archaea, and viruses, respectively, in each of the 10 samples was used. Hooded crane samples and wild duck samples are shown in red and blue, respectively.

To characterize the biome detected in each sample, we performed a principal component analysis (PCA) using the fraction of sequence reads corresponding to the order of the eukaryotes or the genus of bacteria, archaea, and viruses, respectively, in each of the 10 samples. Principal component (PC) 1 and PC2, and their proportions of variance were 0.412 and 0.332, respectively (Fig. 4). PC1 clearly distinguished between the fecal samples of hooded cranes and wild ducks, except for sample #10. We therefore examined the eigenvector of PC1, and found that the contribution of *Bacteroides* or *Solanales* was the largest (0.611 or 0.021, respectively). Indeed, *Bacteroides* or *Solanales* are prominent in duck species (Figs 2 and 4). In contrast, the contribution of *Cetobacterium* or *Clupeiformes* was the smallest (−0.675 or −0.024, respectively), but was prominent in the hooded crane samples (Figs 2 and 4). In addition, the contribution of viruses belonging to the genus *Pbunavirus* was low (−0.043), but was significant in the hooded crane samples (Fig. 2B).

To evaluate the diversity of each sample further, we computed the alpha diversity by using Shannon's Diversity Index. We found no significant differences in alpha diversity between hooded cranes and wild ducks (Tables S7 and S8). To evaluate the similarity of diversity among our samples, we calculated the beta diversity by using the Jaccard Index of Dissimilarity and found that the hooded crane and wild duck fecal samples each exhibited similar diversity within their respective species. (Tables S9–S14). These results suggest that hooded cranes may have their own gut microbiota.

## Discussion

In this study, we analyzed metagenomic sequencing data (65 535 573 reads on average per sample, Table 1 and Fig. 1) of fecal samples from hooded cranes that winter on the Izumi plain in Japan to identify the bacterial and viral species and gut microbiota composition of these birds, and to gain insights into the hooded crane diet. We successfully obtained proportions of organisms for each sample at the order or genus level for eukaryotes or bacteria, archaea, and viruses, respectively; however, it was difficult to identify exact species mainly due to the short length of the reads (150 nt). We therefore conducted sequencing assembly of the short reads for each sample and analyzed the organisms in the samples at the species level (Table S4).

Intestinal microbial communities are affected by the gut anatomy [23]. The PCA results suggested (Fig. 4) differences in the intestinal microbiota contained in the feces of hooded cranes and ducks. The bacterial genus *Bacteroides*, which was detected at relativity high proportions in multiple wild duck samples (except for sample #10) was rarely detected in hooded cranes (Fig. 2A and Table 2). In contrast, the taxa of the genera *Cetobacterium* and *Escherichia* were detected at relativity high proportions in multiple hooded crane samples, but were barely detected in wild ducks (Fig. 2A and Table 2). Wild ducks belong to the genus *Anas*, but our wild duck samples came from different species: #7 and #9 are Eurasian wigeon (*Anas penelope*), #8 is mallard or spot‐billed duck (*Anas platyrhynchos* or *Anas poecilorhyncha*), and #10 is northern pintail (*Anas acuta*) (Table 1). Hence, these results may indicate differences between Eurasian wigeon intestinal microbiota and hooded crane intestinal microbiota. Accumulating data on the intestinal microbiota of waterfowl provides important information for wildlife conservation decisions. As for archaea, we did not see clear diversity among archaeal genera across our sequencing reads (Fig. 2B and Table S3), and we found no genera suggesting a correlation between the results of the bacterial microbiota and viral microbiota of hooded cranes or wild ducks, although many archaea exist in human intestines [24]. There were multiple reads that could not be identified in this study, and they may have included unidentified archaea. In addition, since this study utilized only DNA, it was not possible to analyze the virus microbiota for RNA viruses. More information is needed to reveal the relationship of the gut microbiota to the archaeal and viral genera.

In this study, we estimated the diet of the hooded cranes that winter on the Izumi plain in Japan by examining metagenomic sequencing data from fecal samples. However, since we collected wild waterfowl fecal samples from rice fields, it was not possible to determine whether the cockroaches and flies contained in the feces were eaten by the waterfowl or were trace contaminants from the sample collection site. In fact, the order *Blattodea* was detected in samples #2, #7, #8, and #9, and the order *Diptera* was detected in samples #7, #8, and #9 (Table 4, Tables S4–S6, Fig. 1B, and Fig. S2). These insects gather and collect in feces; therefore, it is possible that their genes were added externally. Furthermore, it is difficult to determine the exact conditions for each sample, such as the length of time between feces discharge and sampling. Therefore, it is not feasible to investigate differences in the proportion of reads across samples. Direct observation of the habitants and/or characterization of digestion in the feces would be needed to clarify this point.

To analyze the interactions and relationships of the various taxa in more detail, we would need to increase the number of samples and accumulate more data. Since wildlife metagenomic data can aid in the discovery and ecology of unknown pathogens that may emerge in the future, it is important to accumulate such data. Overall, this study has revealed the food contents and bacterial and viral microbiota in the gut of hooded cranes that flew to Izumi, Japan. Our findings not only improve our understanding of wild waterfowl ecology, but will also greatly contribute to wildlife conservation efforts.

## Conflict of interest

The authors declare no conflict of interest.

### Peer review

The peer review history for this article is available at https://www.webofscience.com/api/gateway/wos/peer‐review/10.1002/2211‐5463.13881.

## Author contributions

KT, SN, MO, and TW conceived the study. KT, SN and KK analyzed data. KT, SN, and TW wrote the manuscript. All authors read and approved the final manuscript.

## Supporting information


**Fig. S1.** Proportion of the Archaeal and Eukaryotic genera in samples of hooded cranes and wild ducks.
**Fig. S2.** Proportion of major taxa of insect, fish, and plant in the eukaryotes reads identified in each sample.

**Table S1.** Genomes used for the metagenomic searches.
**Table S2.** Percentage of eukaryotes, bacteria, archaea, and viruses in the identified reads.
**Table S3.** Percentage of taxa in the top five of the Archaea reads identified in each sample.
**Table S4.** Species of insects, fish, and plant identified in the assembly data in each sample.
**Table S5.** Percentage of major taxa of insect, fish, and plant in the eukaryotes reads identified in each sample.
**Table S6.** Percentage of taxa in the top five of the eukaryotes reads identified in each sample.
**Table S7.** Species‐level alpha diversity in each sample.
**Table S8.** Genus‐level alpha diversity in each sample.
**Table S9.** Difference in species content for each sample.
**Table S10.** Difference in genus content for each sample.
**Table S11.** Species‐level Jaccard distance for each sample.
**Table S12.** Genus‐level Jaccard distance for each sample.
**Table S13.** Species‐level Bray–Curtis dissimilarity for each sample.
**Table S14.** Genus‐level Bray–Curtis dissimilarity for each sample.

## Data Availability

The data underlying this article will be shared upon reasonable request to the corresponding author. All RNA‐seq data sequenced in this study were deposited in the DDBJ Sequence Read Archive (DRA) with the following accession numbers: DRR173096–DRR173101 and DRR279219–DRR279222. The source code for the diversity calculation is available at https://biokirr.com/Supporting‐Data/Diversity‐Scripts/.
