# Supplementary material for: Metagenomic analysis of the gut microbiota of hooded cranes (Grus monacha) on the Izumi plain in Japan
Source: FEBS Open Bio. 2024 Sep 14;14(12):1972–84. doi: 10.1002/2211-5463.13881 (PMC11609581; doi:10.1002/2211-5463.13881)
Supplement: Supplementary file 1 — Fig. S1. Proportion of the Archaeal and Eukaryotic genera in samples of hooded cranes and wild ducks. Fig. S2. Proportion of major taxa of insect, fish, and plant in the eukaryotes reads identified in each sample. [file FEB4-14-1972-s002.pptx]

## Slide 1
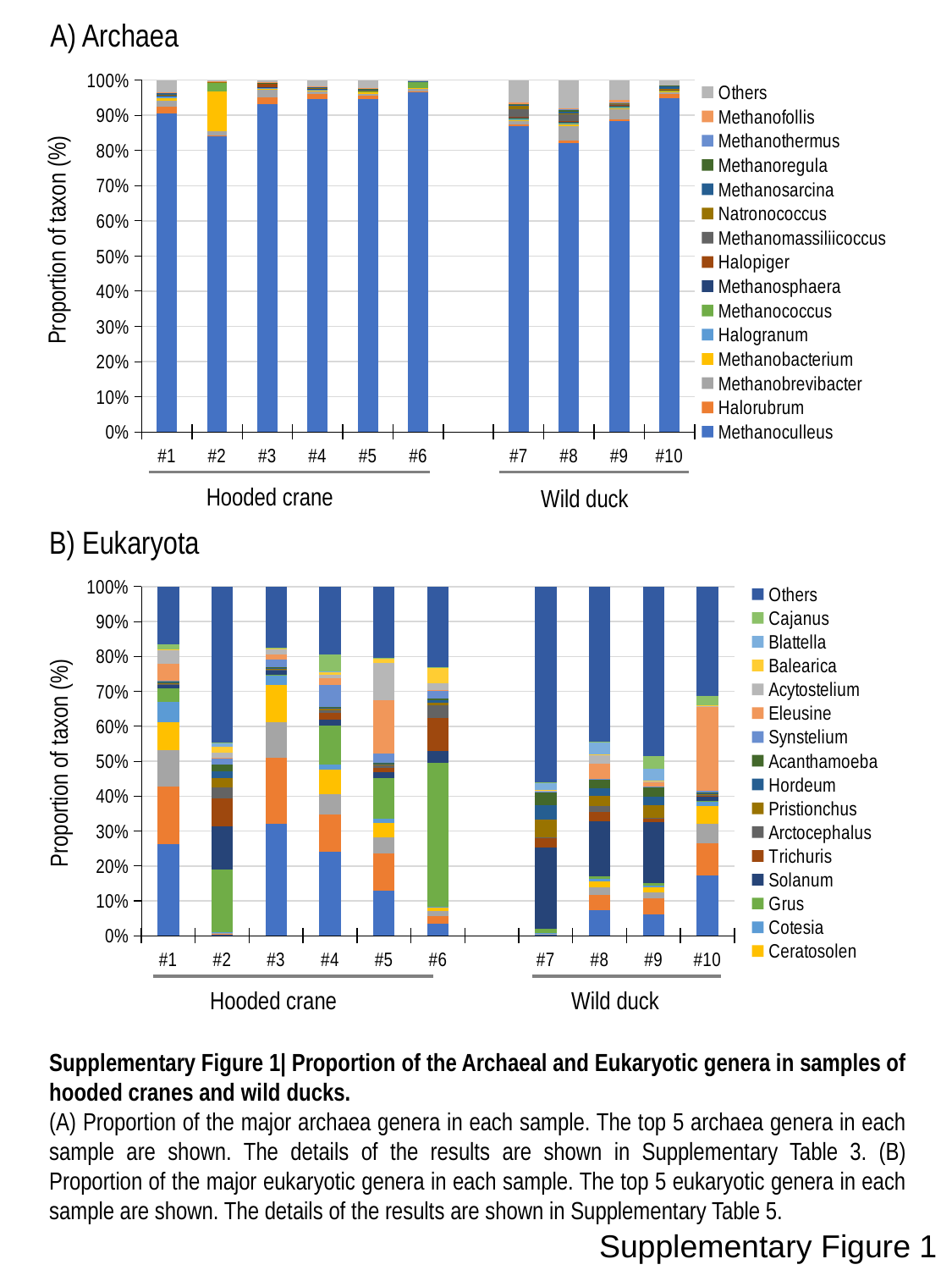

A) Archaea
### Chart
| Category | Methanoculleus | Halorubrum | Methanobrevibacter | Methanobacterium | Halogranum | Methanococcus | Methanosphaera | Halopiger | Methanomassiliicoccus | Natronococcus | Methanosarcina | Methanoregula | Methanothermus | Methanofollis | Others |
|---|---|---|---|---|---|---|---|---|---|---|---|---|---|---|---|
| #1 | 90.44735862701422 | 1.9604564982489372 | 1.7341925282548205 | 0.8080875507726608 | 0.3816623394084067 | 0.1401784526850534 | 0.016491582668829816 | 0.277608302761441 | 0.016491582668829816 | 0.10240487838008279 | 0.3778441092394233 | 0.26386532270127705 | 0.0 | 0.06596633067531926 | 3.4073918945207 |
| #2 | 84.17321365097294 | 0.0150273636766564 | 1.172134366779199 | 11.43582375793552 | 0.0 | 2.5696791887082444 | 0.075136818383282 | 0.0150273636766564 | 0.0150273636766564 | 0.0150273636766564 | 0.07059553405283275 | 0.0300547273533128 | 0.0300547273533128 | 0.0150273636766564 | 0.36817041007807916 |
| #3 | 93.17023557208223 | 1.8441521787024133 | 2.1159339487853943 | 0.5908911325967625 | 0.11436602658619617 | 0.11436602658619617 | 0.028591506646549043 | 1.0936251292305008 | 0.0 | 0.052417752654837696 | 0.12153260209318026 | 0.028591506646549043 | 0.042887259969823566 | 0.028591506646549043 | 0.6538178507728105 |
| #4 | 94.61657736649794 | 1.3423213656700346 | 0.9049702641563245 | 0.3102003182027418 | 0.10810010712883039 | 0.11275545814427829 | 0.03384003471302638 | 0.21996022563467144 | 0.022560023142017584 | 0.0639200580490421 | 0.37029418504688605 | 0.07896008099706155 | 0.011280011571008792 | 0.06016005795204303 | 1.744100443094112 |
| #5 | 94.65274392232585 | 0.9079386917932432 | 0.5285126468289078 | 0.41959200221061593 | 0.22792652489949514 | 0.02072058752161064 | 0.0 | 0.17094488978951022 | 0.03108088905263822 | 0.12432354067010835 | 0.08252815227479868 | 0.49729422484221153 | 0.0 | 0.023310666789478667 | 2.3130832610015233 |
| #6 | 96.56299329453304 | 0.18474664727881157 | 0.9158630452849065 | 0.04016231462582861 | 0.0 | 1.906371206260973 | 0.03212985170066288 | 0.01606492585033144 | 0.01606492585033144 | 0.024097388775497164 | 0.038876365506287126 | 0.05622724047616005 | 0.12851940680265153 | 0.0 | 0.07788338705451281 |
| | None | None | None | None | None | None | None | None | None | None | None | None | None | None | None |
| #7 | 86.92424985349135 | 0.37807380225037096 | 1.1344834410959264 | 0.10960397047103006 | 0.5218167143435251 | 0.07190148034734055 | 0.08088541235316268 | 0.30208471369576934 | 2.0782829343522105 | 0.9808956398410362 | 0.22153318917560305 | 0.2245983001455534 | 0.008983932005822136 | 0.5090181778562348 | 6.453588438575068 |
| #8 | 82.01184823426642 | 0.7307444553163062 | 4.255945397808309 | 0.4161134733005648 | 0.2528284394737609 | 0.1951234453122585 | 0.14771811291092837 | 0.23483196366876224 | 1.927816850987427 | 0.25610166179637134 | 0.4646574607171383 | 0.674209171930029 | 0.03160355493422011 | 0.4018166360503857 | 7.998641141527116 |
| #9 | 88.42700425962298 | 0.45058199747839794 | 2.859853230981812 | 0.19404131832779603 | 0.2278857486400485 | 0.2573382702140762 | 0.17611166995531038 | 0.19940002667560075 | 0.48735960155259467 | 0.21226090504990533 | 0.05505358913241905 | 0.15568431716263442 | 0.013537766709794296 | 0.5221065270825556 | 5.761780771414095 |
| #10 | 94.9008980785818 | 1.1666387575786132 | 0.6371005981103065 | 0.10756243864199981 | 0.13514254008226245 | 0.10761669975527782 | 0.0 | 0.07170828691197738 | 0.0 | 0.38060555211784547 | 0.9406931982556334 | 0.04964420245015375 | 0.0 | 0.0 | 1.5023896475141356 |Proportion of taxon (%)
Hooded crane
Wild duck
B) Eukaryota
### Chart
| Category | Tenualosa | Plutella | Pterocnemia | Ceratosolen | Cotesia | Grus | Solanum | Trichuris | Arctocephalus | Pristionchus | Hordeum | Acanthamoeba | Synstelium | Eleusine | Acytostelium | Balearica | Blattella | Cajanus | Others |
|---|---|---|---|---|---|---|---|---|---|---|---|---|---|---|---|---|---|---|---|
| #1 | 26.26533083128696 | 16.45020935265916 | 10.410705327797034 | 8.109466977558407 | 5.848347811782983 | 3.7208251691106096 | 1.2427569104210774 | 0.0951160905844651 | 0.04241604130373138 | 0.2354506438238088 | 0.27462463377533924 | 0.18129357887917158 | 0.21749683287610747 | 4.695371251697123 | 3.9466253261954756 | 0.37091026950416467 | 0.0014301892082057262 | 1.2839210425436303 | 16.60770171899253 |
| #2 | 0.3590761106807789 | 0.2588956913282417 | 0.23770758922728344 | 0.16696117015662754 | 0.04184980168539693 | 17.865228747765812 | 12.347511633481968 | 7.951435315131435 | 3.321479887284372 | 2.5602282408693986 | 2.0984002537677413 | 1.8071039084511056 | 1.731949217613588 | 0.14286393802447847 | 1.4829112997529366 | 1.779978947054552 | 1.0510420314291378 | 0.10360501512148826 | 44.69177120117366 |
| #3 | 32.11151377338872 | 18.783173756758224 | 10.218301203206382 | 10.74843324591774 | 2.5544955695650335 | 0.4158496833926771 | 1.3346903640357015 | 0.04559860008853164 | 0.015403408296086281 | 0.25097614541564345 | 0.19861044271397071 | 0.19028236266282048 | 2.2018991804937804 | 1.5553383762164223 | 1.551567648975654 | 0.03372467429581521 | 0.002317166662048303 | 0.30852105610360964 | 17.479303341811132 |
| #4 | 24.051399869081546 | 10.736846282333538 | 5.709267358389292 | 7.174202433552811 | 1.3592948499294215 | 11.14069585373198 | 1.6939423223736199 | 1.9859234273519315 | 0.7981088001715684 | 0.3794376646247359 | 0.2927694238900459 | 0.25908986372124254 | 6.321730699997088 | 1.8073613923958773 | 1.0201529312094673 | 1.088624090166052 | 0.002572522033881962 | 4.761740685882604 | 19.416839529163298 |
| #5 | 12.946478765895385 | 10.643916743190774 | 4.54014783892834 | 4.1704205461591135 | 1.2822618685744676 | 11.605996009229486 | 1.6059261825602518 | 1.2533870922523744 | 0.7130753077871613 | 0.34987841986678875 | 0.27383721862601523 | 0.24934699556109066 | 2.446222933753092 | 15.328775279531381 | 10.755988111550055 | 1.12541318047856 | 0.004148275661625422 | 0.3714874499561957 | 20.333291780437847 |
| #6 | 3.5272233129347392 | 2.040864142736572 | 1.5871427698426703 | 0.8260048385800942 | 0.41456076287875177 | 41.190538140280594 | 3.2336727905298597 | 9.480313153666385 | 3.761456032140071 | 0.7879718764451841 | 0.617829426353351 | 0.469599743037927 | 2.2306188996563376 | 0.19541596757439322 | 1.9072432215895823 | 4.486625852449697 | 0.08226949133277721 | 0.060500867370362 | 23.10014871060065 |
| | None | None | None | None | None | None | None | None | None | None | None | None | None | None | None | None | None | None | None |
| #7 | 0.3483805406608942 | 0.1965459332252391 | 0.12524249185476116 | 0.11072852693274145 | 0.03674270867602673 | 1.1769944194673139 | 23.3979071285394 | 2.7578352100073826 | 0.014019353552756965 | 5.016585238240228 | 4.3059255088942665 | 3.803910569325596 | 0.06096828606166974 | 0.216074615458887 | 0.06302976248909516 | 0.016856737476385438 | 2.1635110723967674 | 0.03349639143073445 | 56.15524550530985 |
| #8 | 7.336415959205642 | 4.458173851820512 | 1.9653342665675946 | 1.8764062579059337 | 0.6786891707390558 | 0.7377440619262275 | 15.810784705305943 | 2.4733188912261546 | 1.810230326185933 | 2.88677716567499 | 2.3168264691124327 | 2.1949319953288144 | 0.3379269984335583 | 4.310333896557392 | 2.7199434278841044 | 0.016843861041432602 | 3.616364246055769 | 0.063714748113699 | 44.38923970091481 |
| #9 | 6.230500673553545 | 4.54189496435048 | 1.6226385395721403 | 1.4091999115442726 | 0.4644988376482009 | 0.8600239639894754 | 17.484216585583752 | 1.1137662472641336 | 0.13823490910009886 | 3.412774125534816 | 2.6517436560033736 | 2.592622890469272 | 0.12883877956653547 | 1.3643420278312506 | 0.42552236621730816 | 0.01742721936897181 | 3.385476874706802 | 3.526360467630106 | 48.62991696006547 |
| #10 | 17.18575269319473 | 9.29285234633413 | 5.515049866042821 | 5.231222981094179 | 1.251895907179129 | 0.15337832904609836 | 1.3807956484045687 | 0.15974253901764132 | 0.07204317098467766 | 0.30391904240917683 | 0.3481041914262644 | 0.23207610265795586 | 0.3422942821024567 | 24.02283786032519 | 0.6283387130081752 | 0.009309614385663302 | 0.004655967577793291 | 2.6124253389681202 | 31.253305405841232 |Proportion of taxon (%)
Hooded crane
Wild duck
Supplementary Figure 1| Proportion of the Archaeal and Eukaryotic genera in samples of hooded cranes and wild ducks.
(A) Proportion of the major archaea genera in each sample. The top 5 archaea genera in each sample are shown. The details of the results are shown in Supplementary Table 3. (B) Proportion of the major eukaryotic genera in each sample. The top 5 eukaryotic genera in each sample are shown. The details of the results are shown in Supplementary Table 5.
Supplementary Figure 1

## Slide 2
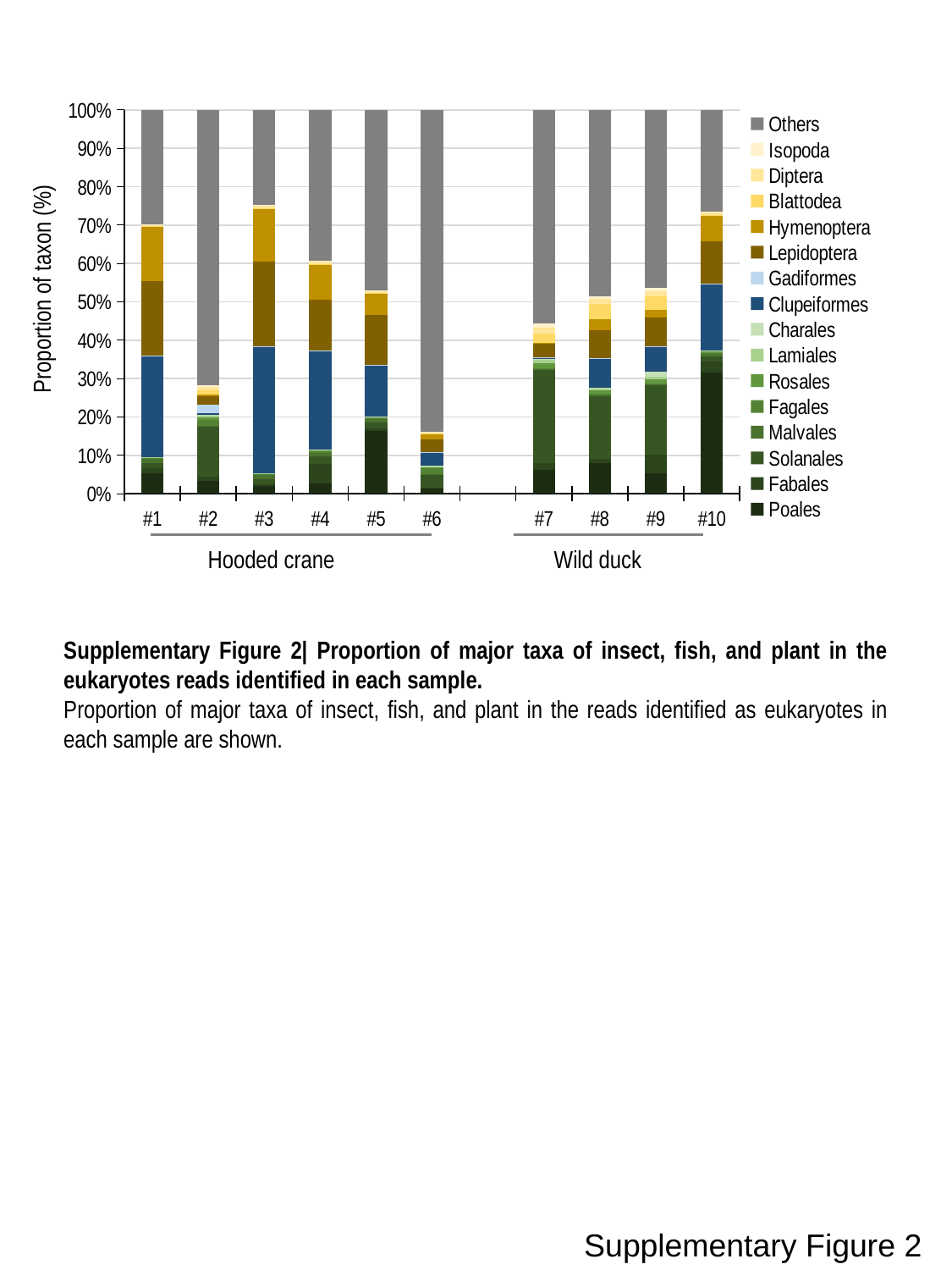

### Chart
| Category | Poales | Fabales | Solanales | Malvales | Fagales | Rosales | Lamiales | Charales | Clupeiformes | Gadiformes | Lepidoptera | Hymenoptera | Blattodea | Diptera | Isopoda | Others |
|---|---|---|---|---|---|---|---|---|---|---|---|---|---|---|---|---|
| #1 | 5.350692434 | 1.384877741 | 1.258518642 | 1.189860228 | 0.05936254 | 0.186186545 | 0.078843582 | 0.002377987 | 26.46603929 | 0.020218723 | 19.45262171 | 14.19193789 | 0.010275405 | 0.564495245 | 0.052261768 | 29.73143026999999 |
| #2 | 3.386702668 | 1.048660284 | 13.1332104 | 0.034294094 | 1.578747229 | 0.732986133 | 0.548036512 | 0.1082424 | 0.413361808 | 2.238176027 | 2.125258679 | 0.404460871 | 1.122299529 | 0.860129965 | 0.597726611 | 71.66770679 |
| #3 | 1.928138745 | 0.421026145 | 1.374938862 | 1.264984932 | 0.031903345 | 0.314285019 | 0.061002168 | 0.001286085 | 32.95393707 | 0.074340937 | 22.10751465 | 13.8656687 | 0.023643536 | 0.706097551 | 0.055274271 | 24.815957984000008 |
| #4 | 2.563396385 | 5.244720393 | 1.831005242 | 0.996156921 | 0.509977103 | 0.281260226 | 0.099815713 | 0.015672618 | 25.76406784 | 0.021375919 | 13.31507088 | 9.274739595 | 0.0082928 | 0.684340761 | 0.075029499 | 39.315078105 |
| #5 | 16.41515796 | 0.526466429 | 1.667820256 | 0.848738849 | 0.159248349 | 0.360161522 | 0.123884901 | 0.012257527 | 13.33728428 | 0.096585814 | 12.94464458 | 5.775645046 | 0.012631384 | 0.556506591 | 0.069189016 | 47.093777495999994 |
| #6 | 1.30366994 | 0.29984629 | 3.365348381 | 0.143916716 | 1.611372106 | 0.273243648 | 0.146377263 | 0.072815146 | 3.634066196 | 0.016249688 | 3.305589038 | 1.320316892 | 0.112096804 | 0.376397347 | 0.136141058 | 83.882553487 |
| | None | None | None | None | None | None | None | None | None | None | None | None | None | None | None | None |
| #7 | 6.153453807 | 1.855970326 | 24.48311937 | 0.036459896 | 0.041169459 | 1.383698073 | 1.051327735 | 0.010374575 | 0.381070569 | 0.15604236 | 3.563429233 | 0.211536014 | 2.334477487 | 1.692227912 | 1.061513854 | 55.58412933 |
| #8 | 7.887735011 | 1.118910951 | 16.44033559 | 0.313587878 | 0.184025788 | 0.930720234 | 0.663564885 | 0.035498284 | 7.638800864 | 0.119476646 | 7.323759896 | 2.82129663 | 3.898930172 | 1.414710657 | 0.653145881 | 48.555500633 |
| #9 | 5.40400574 | 4.863331124 | 18.04312906 | 0.291635466 | 0.073316755 | 1.048343867 | 0.746513173 | 1.411534526 | 6.447018095 | 0.130638514 | 7.455486917 | 2.023465035 | 3.581212189 | 1.349526033 | 0.759561268 | 46.371282238000006 |
| #10 | 31.56227602 | 2.83617936 | 1.42857144 | 0.714841634 | 0.133256174 | 0.451094799 | 0.161947244 | 0.002436699 | 17.34457383 | 0.01872173 | 11.21213141 | 6.754006021 | 0.030770084 | 0.72684471 | 0.062107844 | 26.56024100100001 |Proportion of taxon (%)
Hooded crane
Wild duck
Supplementary Figure 2| Proportion of major taxa of insect, fish, and plant in the eukaryotes reads identified in each sample.
Proportion of major taxa of insect, fish, and plant in the reads identified as eukaryotes in each sample are shown.
Supplementary Figure 2
